# The prominent role of the S100A8/S100A9-CD147 axis in the progression of penile cancer

**DOI:** 10.3389/fonc.2022.891511

**Published:** 2022-10-11

**Authors:** Tobias Mohr, Anabel Zwick, Muriel Charlotte Hans, Isabelle Ariane Bley, Felix Leon Braun, Oybek Khalmurzaev, Vsevolod Borisovich Matveev, Philine Loertzer, Alexey Pryalukhin, Arndt Hartmann, Carol-Immanuel Geppert, Hagen Loertzer, Heiko Wunderlich, Carsten Maik Naumann, Holger Kalthoff, Kerstin Junker, Sigrun Smola, Stefan Lohse

**Affiliations:** ^1^ Institute of Virology, Saarland University Medical Center, Homburg, Germany; ^2^ Department of Urology and Paediatric Urology, Saarland University Medical Center, Homburg, Germany; ^3^ Department of Urology, Federal State Budgetary Institution “N.N. Blokhin National Medical Research Center of Oncology” of the Ministry of Health of the Russian Federation, Moscow, Russia; ^4^ Department of Urology and Paediatric Urology, Westpfalz Klinikum, Kaiserslautern, Germany; ^5^ Institute of Pathology, Saarland University Medical Center, Homburg, Germany; ^6^ Institute of Pathology, University Medical Center Bonn, Bonn, Germany; ^7^ Institute of Pathology, University Erlangen-Nuremberg, Erlangen, Germany; ^8^ Department of Urology and Paediatric Urology, St. Georg Klinikum, Eisenach, Germany; ^9^ Department of Urology and Paediatric Urology, University Hospital Schleswig Holstein, Kiel, Germany; ^10^ Division of Molecular Oncology, Institute of Experimental Cancer Research, University Hospital Schleswig Holstein, Kiel, Germany

**Keywords:** penile cancer, myeloid-derived suppressor cells, neutrophils, calprotectin, HPV-related cancer, S100A8, S100A9, CD147

## Abstract

Currently, no established biomarkers are recommended for the routine diagnosis of penile carcinoma (PeCa). The rising incidence of this human papillomavirus (HPV)–related cancer entity highlights the need for promising candidates. The Calprotectin subunits S100A8 and S100A9 mark myeloid-derived suppressor cells in other HPV-related entities while their receptor CD147 was discussed to identify patients with PeCa at a higher risk for poor prognoses and treatment failure. We thus examined their expression using immunohistochemistry staining of PeCa specimens from 74 patients on tissue microarrays of the tumor center, the invasion front, and lymph node metastases. Notably, whereas the tumor center was significantly more intensively stained than the invasion front, lymph node metastases were thoroughly positive for both S100 subunits. An HPV-positive status combined with an S100A8^+^S100A9^+^ profile was related with an elevated risk for metastases. We observed several PeCa specimens with S100A8^+^S100A9^+^-infiltrating immune cells overlapping with CD15 marking neutrophils. The S100A8^+^S100A9^+^CD15^+^ profile was associated with dedifferentiated and metastasizing PeCa, predominantly of HPV-associated subtype. These data suggest a contribution of neutrophil-derived suppressor cells to the progression of HPV-driven penile carcinogenesis. CD147 was elevated, expressed in PeCa specimens, prominently at the tumor center and in HPV-positive PeCa cell lines. CD147^+^HPV^+^ PeCa specimens were with the higher-frequency metastasizing cancers. Moreover, an elevated expression of CD147 of HPV-positive PeCa cell lines correlated negatively with the susceptibility to IgA-based neutrophil-mediated tumor cell killing. Finally, stratifying patients regarding their HPV/S100A8/S100A9/CD15/CD147 profile may help identify patients with progressing cancer and tailor immunotherapeutic treatment strategies.

## Introduction

The tumor microenvironment (TME) displays a critical and fate-deciding compartment of neoplasia (1). It has the potential to drive a cancer-related inflammation that unremittingly recruits innate immune cells, such as macrophages and neutrophils (1–3). Once trapped inside the tumor, the TME reprograms myeloid cells to tumor-associated macrophages, tumor-associated neutrophils (TANs), or myeloid-derived suppressor cells (MDSCs) (1–3). These contribute to tumor progression at all steps of carcinogenesis, from initiation over the progression of precursor lesions to invasive growing cancers, the dissemination of cancer cells, metastatic outbreaks, and preparation of a pre-metastatic niche to facilitate the seeding at distant anatomical sites (1, 4, 5). An elevated infiltration with unfavorable neutrophils counts to the most negative risk factors for the most dismal prognosis for patients with cancer (6, 7). Moreover, by expressing immune checkpoints, TANs and MDSCs can provoke resistance to immunotherapeutic approaches, especially regarding T-cell–based strategies (1, 8, 9). Thus, markers for these myeloid cells have the potential to identify patients at high risk for worse prognosis and may have a decision-making impact on subsequent therapeutic interventions.

Penile carcinoma (PeCa) displays a rare disease with notable regional differences regarding the incidence rates (10–12). Patients with PeCa present with histological and pathogenic heterogeneous cancers, with penile squamous cell carcinoma (PSCC) as the most common histological subtype. Penile carcinogenesis is frequently linked to chronic infections with human papillomavirus (HPV) as a prominent etiologic agent (13). A recent meta-analysis on global data calculated an overall HPV prevalence of 50.8% with HPV16 as the predominant HPV type in PeCa (14). Although the subtype classification seems to have prognostic potential, this is controversially discussed for HPV (14, 15). Currently, especially advanced or metastatic PeCa still has a poor prognosis and is a therapeutic challenge with limited options. This further highlights the need for new markers identifying patients with PeCa subtypes that display a high risk for advanced and metastasizing cancers, allowing a more precise stratification for more aggressive or de-escalated treatment regimes.

We recently described CD15, a surface protein of neutrophils, as a promising candidate and identified an HPV^active^p63^high^CXCL8^high^ axis as a possible underlying mechanism that drives neutrophil infiltration (16). Subsequent inclusion of clinic-pathological data revealed that especially HPV^+^p63^+^CD15^+^ PeCa was characterized by dedifferentiation, invasion, and metastases. Earlier data pointed to a role for the Calprotectin subunits S100A8 and S100A9 in the chemotaxis of neutrophils in the HPV-associated malignant transformation of the skin (17, 18). Although viral oncoproteins of cutaneous and mucosal HPV types may cause, in part, similar but not the same molecular alterations (18, 19), we hypothesized that the Calprotectin subunits S100A8 and S100A9 play a role in the mucosal high-risk (HR) HPV–driven malignant transformation as well. The current literature provides initial evidence for the Calprotectin subunits driving progression and chemoresistance of HPV-associated cancers of other anatomical sites (20–22) and to contribute already to early events in HPV-driven carcinogenesis (23). Moreover, S100A8 and S100A9 mark MDSCs critically involved in the mobilization of further neutrophils in the pre-metastatic niche, facilitating the arrival and survival of metastatic cells (1–3). The infiltration of immunosuppressive myeloid cells has been demonstrated in a mouse model for penile cancer, mimicking HPV-driven carcinogenesis (24). Targeting MDSCs and TME-related myeloid cell reprogramming significantly increased the efficacy of the immune checkpoint antibody-based therapy in this mouse model.

The S100A8/S100A9 heterodimer Calprotectin can bind to Emmprin, alias CD147 or Basigin, with the S100A9 subunit having a higher affinity than S100A8 (21, 25, 26). CD147 can serve as a receptor for Calprotectin on tumor cells, neutrophils, and fibroblasts. Its activation leads to activated mitogen-activated protein (MAP) kinase and Nuclear factor kappa B (NFkB) pathways, resulting in the upregulation of genes involved in cell survival, proliferation, extracellular matrix remodeling, and inflammation (27, 28). CD147 acts on fibroblasts by stimulating the release of matrix metalloproteases (MMPs), thereby creating a positive feedback loop fueling the growth, angiogenesis, and dissemination of tumor cells (29–31). CD147 as membranous and soluble version and with its potential of cis-, trans-, and homotypic interactions displays a central player in tumor progression and predicts a high risk for malignant progression and chemotherapy resistance (32). Thus, there is a strong rationale to decipher the expression and relevance of the S100A8/S100A9-CD147 axis in PeCa.

Current epidemiologic data on PSCC imply a rising incidence of this HPV-associated malignant disease (33–35). These data further underline the need for promising markers, especially of MDSCs, because our recently published data already suggested the involvement of myeloid cells during HPV-driven penile carcinogenesis (16, 36). Tissue microarrays (TMAs) with a high number of clinical PeCa specimens with matching clinic-pathological data were used to identify a promising candidate marker profile of patients at a higher risk for lymph node involvement. A set of unique HPV-positive PeCa cell lines was used to investigate the expression of CD147 and detected a reciprocal pattern compared to the susceptibility of these tumor cells for IgA antibody-dependent neutrophil-mediated tumor cell killing.

## Materials and methods

### Ethical statement, cohort and study design, and material identifiers

The local Ethics Committee of the Saarland (Ärztekammer des Saarlandes, Saarbrücken, Germany) in accordance with the Declaration of Helsinki approved the experiments with human material used in this study, and a written consent form was provided by the study participants. The TMA cohort consists of patients derived from Russia and Germany between 1992 and 2015. Data on clinical outcome and HPV status were published previously (15, 16). Briefly, DNA was isolated from formalin-fixed paraffin-embedded (FFPE) tissue sections by the QIAamp DNA FFPE Tissue Kit (Qiagen, Hilden, Germany) following the manufacturer’s protocol, and the HPV PCR was conducted using the GP5+/6+ primers as described previously (15, 16). HPV status was further determined by p16^INK4A^ immunohistochemistry (IHC), a surrogate marker for active HPV oncoprotein–driven transformation, using a published protocol (15, 16). HPV status was considered as positive in case of positive PCR and IHC with subsequent genotyping using INNO-LiPA genotyping extra II (Fujirebio Germany GmbH, Hannover, Germany), revealing HPV16, HPV18, and HPV59 in 27 (93.1%), one (3.45%), and one (3.45%) of the 29 cases, respectively. Sections of all cases were reviewed by two experienced uropathologists, and histological subtypes and tumor grade were defined according to the 2016 WHO classification and the eighth editions of TNM classification of malignant tumors. Specimens were stratified as non-invasive (pTis and pT1a) and invasive (pT1b, pT2, pT3, and pT4), not metastasizing (cN0 and pN0) and metastasizing (pN1, pN2, and pN3). Basic cohort description and patient/disease characteristics are listed in **Supplementary Table 1**. Of the 74 patients in total, respective tissue was punched for the individual TMA reflecting tumor center (TMA TC), invasion front (IF), lymph node metastases (TMA LM), and adjacent normal tissue (TMA NO) as duplicates (TMA TC, IF, and NO) and triplicates (TMA LM) with the total number of evaluable specimens indicated for each staining.

### Cell lines and culture conditions

Three HPV-positive PeCa cell lines were generated previously from a primary carcinoma and lymph node metastases, including the particularly rare case of one primarius-derived (named P2) and one metastasis-derived (named L2) cell line originating from the same patient and one further metastasis-derived cell line of an additional patient (named L3). The cell lines were authenticated by Multiplexion in 2018 using the originating biopsies obtained from patients that underwent penectomy and metachronous radical inguinal lymph node dissection for metastatic squamous cell carcinoma of the penis at the University Hospital Schleswig-Holstein, thus representing a validated cell culture system (37). The HPV status of the cell lines was investigated recently (16). Cells were cultivated in PeCa medium {1:1 mixture of keratinocyte growth medium 2 (KGM2) containing all supplements [C-20011, bovine pituitary extract at 0.004 ml/ml, epidermal growth factor (EGF) at 0.125 ng/ml, insulin at 5 µg/ml, hydrocortisone at 0.33 µg/ml, epinephrine at 0.39 µg/ml, transferrin at 10 µg/ml, calcium chloride at 0.06 M; PromoCell, Heidelberg, Germany] and RPMI 1640 containing 10% heat-inactivated fetal bovine serum (FCS), 1% sodium pyruvate, and 1% penicillin and streptomycin (R10+/+, Merck, Schnelldorf, Germany)}. Normal foreskin keratinocytes (NFKs) and human foreskin fibroblasts (HFFs) were isolated from foreskin tissue (Saarland University Medical Center), tested negative for HPV using PCR, and expanded in KGM2 (C-20011, PromoCell) and Dulbecco's Modified Eagle's Medium (DMEM) with 10% heat-inactivated FCS, 1% sodium pyruvate, and 1% penicillin and streptomycin (D10+/+), respectively. NFKs were cultured in PeCa medium for experiments. We conducted mycoplasma-specific PCRs on a regularly basis of once per month. The human vulva carcinoma cell line A431 (DSMZ, Braunschweig, Germany, ACC-91, obtained 2018, RRID : CVCL_0037) was cultured in R10+/+. Cell lines were used below passage 20, NFKs up to passage 4, and HFFs up to passage 7. Organotypic three-dimensional (3D) cultures were generated using HFF (5 × 10^5^ cells, passages 3–5) embedded in 1 ml of rat collagen [as described previously (16)] in 24-well plate in D10+/+. The day after, medium was exchanged to PeCa medium for 1 h before 7 × 10^5^ PeCa cells were seeded on top of the collagen-fibroblast matrix. The next day, the cultures were transferred onto a metal grid in six-well plates to allow multilayered growth at the air-liquid interface. Fourteen days later, supernatants were harvested, and organotypic 3D cultures were fixed in 4% paraformaldehyde (Merck) and embedded in paraffin.

### Antibodies

The engineered 225-IgA2m(1)-N166G-P221R-C331S-N337T-I338L-T339S-dC471-dY472 further named 225-IgA2.0 was produced using the variable regions of the m225 antibody and the engineered IgA2m(1) constant region as previously described (38). ChromPure human Serum IgA purchased from the Jackson ImmunoResearch Labs (#009-000-011, RRID : AB_2337047) served as IgA isotype control.

### Calcein assay

Target cells (2 × 10^4^) per well were labeled for 30 min at 37°C with 10 μM Calcein-AM (Fisher Scientific, C3099) in suspension. Effector cells were isolated as previously described (39). After washing three times, isolated effector cells were added with an effector-to-target cell ratio (E:T ratio) of 40:1. Antibodies were added to the microtiter plates in triplicates as indicated. For maximum release, labeled target cells were treated with 1% Triton X-100 (Sigma-Aldrich) or left untreated for basal release. After 3 h of incubation at 37°C, plates were centrifuged; supernatants were transferred into black 96-well plates with clear flat bottom (Sigma-Aldrich), and fluorescence was measured in the Victor II plate reader (PerkinElmer, Waltham, MA, USA). Percentage of cellular cytotoxicity was calculated using the equation: % specific lysis = (experimental release − basal release)/(maximal release − basal release). Antibody-independent cytotoxicity (effectors without target antibodies) or effector-independent cytotoxicity (target antibodies without effectors) was not observed.

### Flow cytometry, ELISA

For CD147 surface expression analysis, cells were seeded and grown to 80% confluence, trypsinized, and stained with mouse anti-human CD147-specific antibody (50 µg/ml; #306202, RRID : AB_314586, BioLegend, Amsterdam, Netherlands) per 150,000 cells for 30 min on ice. After washing, bound antibody was stained with mouse-immunoglobulin G kappa chain (IgGκ) phycoerythrin (PE)-conjugated binding protein (sc-516141, Santa Cruz, Dallas, TX, USA) and measured with a FACS Canto II (BD Biosciences). Relative fluorescence intensity (RFI) was calculated using the following equation: mean fluorescence intensity specific Ab/mean fluorescence intensity control Ab. Soluble CD147 was quantified in 3D culture supernatants using Human CD147/EMMPRIN ELISA (#ELH-CD147-1, RayBiotech, Peachtree Corners, USA) according to the manufacturer’s instructions.

### Immunohistochemistry

formalin-fixed paraffin-embedded (FFPE) tissue (TMA) slides were stained by IHC. Antigen retrieval was performed by heating the sections in 1 mM citrate buffer (pH 6.0) at 95˚C for 10 min, and endogenous peroxidase activity was blocked with 3% H_2_O_2_/tris buffered saline (TBS) for 10 min [for 3,3`-Diaminobenzidine (DAB) staining]. formalin-fixed paraffin-embedded (FFPE) sections were incubated with the S100A8-specific (#NBP1-42076, rabbit anti-human S100A8, RRID : AB_2184111, Novus Biologicals, Cambridge, UK), S100A9-specific (#sc-20173, rabbit-anti-human S100A9, RRID : AB_2184420, Santa Cruz, Heidelberg, Germany), or CD147/Emmprin-specific (BioLegend, Cat# 306202, RRID : AB_314586) antibodies overnight followed by Alkaline phosphatase/horseradish peroxidase (AP/HRP-conjugated anti-rabbit/mouse antibody incubation and developed with 3,3`-Diaminobenzidine (DAB) or HRP substrates [ImmPRESS HRP/Peroxidase Reagent Kit and VECTOR Red Alkaline Phosphatase Substrate Kit (Alkaline Phosphatase Anti-Rabbit/Mouse IgG), both Vector, Burlingame, CA, USA]. After counterstaining with Hematoxylin Nuclear Counterstain (Gill’s Formula) (Vector), the slides were covered with Vectamount and documented. Staining was documented using a Leica DMI6000 (RRID : SCR_018713) with LAS X software (RRID : SCR_013673).

### Data processing and statistical analyses

Graphical, correlation, and statistical analyses were performed using GraphPad Prism 9.02 (GraphPad Software, San Diego, CA, USA, RRID : SCR_002798). The annual percentage change (HRPC) was calculated using the following formula: HRPC = {Exp(slope) − 1} × 100. Group data are reported as mean ± SEM. Data of multiple experiments were illustrated as a box and whisker plot showing individual results with minimum and maximum values. Significance was determined by two-way or one-way ANOVA repeated measures test with Tukey’s correction or Fisher’s exact test as indicated, and odds ratios were calculated using the Baptista-Pike method. Significance was accepted when p-values were ≤ 0.05.

## Results

### S100A8 and S100A9 mark tumor centers and lymph node metastasis

**Figure 1:**
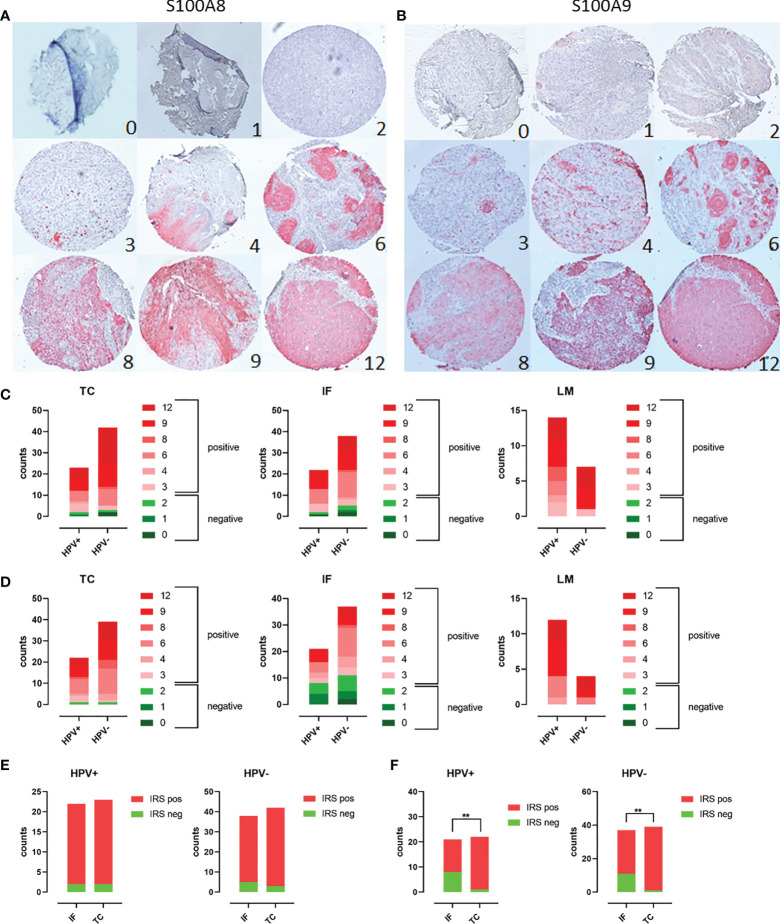
Scoring of PeCa specimens stained using IHC for S100A8/A9: TMAs containing PeCa specimens were stained for S100A8 **(A)** and S100A9 **(B)**. According to Remmele and Stegner, IRSs were defined according to staining intensity and the number of stained cells per specimen. IRSs ≥ 3 were considered positive and below 3 as negative for S100A8/S100A9. Cases per IRS were counted for HPV^−^ and HPV^+^ PeCa specimens on the TC, IF, and LM TMA regarding S100A8 **(C)** and S100A9 **(D)**. Counts of specimens with positive and negative IRS for S100A8 **(E)** and S100A9 **(F)** for HPV^+^ and HPV^−^ specimens comparing the invasion front (IF) and tumor center (TC). Significant differences are indicated with asterisk for p ≤ 0.01 (**0.0093) as calculated by the Fisher’s exact test.

The expression of both Calprotectin subunits S100A8 and S100A9 was examined in clinical PeCa specimens using three TMAs reflecting the tumor center (TMA TC, n = 63), the invasion front (TMA IF, n = 57), and the corresponding lymph node metastases (TMA LM, n = 22). The HPV^+^ status was defined as positive for GP PCR and p16^INK4A^ IHC (15). Slides were stained by IHC and immune reactive scores (IRSs) were determined according to Remmele and Stegner (**Figures 1A, B**). Tumor nests and infiltrating immune cells were intensively stained, whereas, in some cases, the tumor margin was spared (**Supplementary Figures 1**, **2**). Staining for both subunits was limited to suprabasal rather superficial layers in the squamous epithelium of non-malignant tissue (**Supplementary Figures 1**, **2**). Specimens with a throughout S100A8^+^/S100A9^+^-positive epithelium or PeCa tissue were identified for each TMA. The majority of PeCa specimens were positive for S100A8 and S100A9 regarding TMA TC (S100A8, 92.1%; S100A9, 91.5%), TMA IF (S100A8, 91.2%; S100A9, 65.5%), and especially of the TMA LM (S100A8, 100.0%; S100A9, 100.0%) (**Figures 1C, D**). Notably, the majority of S100A8^+^S100A9^+^ specimens were HPV^−^ for the TMA TC (S100A8, 63.5%; S100A9, 62.7%) and IF (S100A8, 61.4%; S100A9, 61.8%), which flips regarding TMA LM (S100A8, 70.0%; S100A9, 80.0%). There was a remarkable and significant increase of positive IRS regarding the tumor center compared to the invasion front for both HPV^+^ (p = 0.0093) and HPV^−^ (p = 0.0013) PeCa for the Calprotectin subunit S100A9 but not for S100A8 (**Figures 1E, F**). The expression of both Calprotectin subunits suggests a TME actively recruiting myeloid cells that once trapped polarizes them into MDSCs (1–3). Of those, especially neutrophil-MDSCs have been repeatedly reported as a negative prognostic marker for patients with dismal prognoses (2, 7, 40–43).

### HPV-positive PeCa specimens are characterized by S100A8^+^S100A9^+^CD15^+^ immune cells

**Figure 2:**
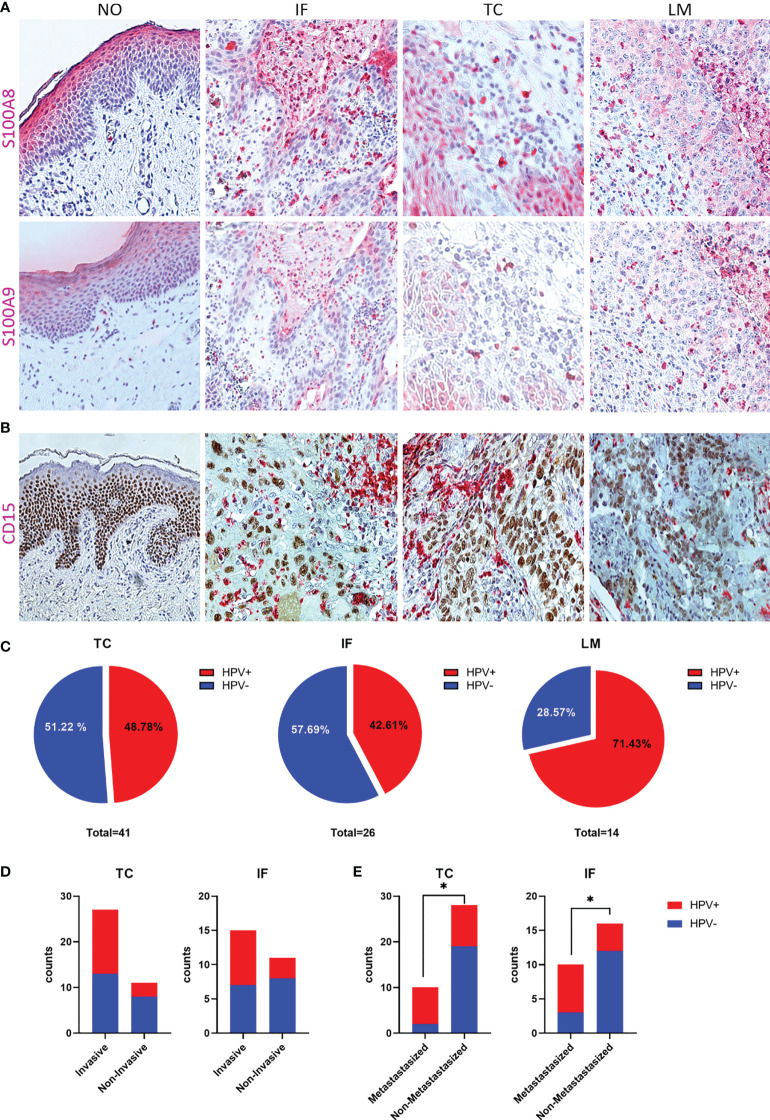
S100A8/A9 is associated with HPV-positive, invasive, metastasizing PeCa with infiltrating neutrophils. **(A)** IHC for S100A8 and S100A9 of normal tissue (NO), and PeCa specimens of the invasion front (IF), tumor center (TC), and lymph node metastases (LM), illustrating S100A8+S100A9+-infiltrating immune cells. **(B)** IHC for CD15 and p63 to illustrate infiltration of neutrophils in PeCa specimens (16). **(C)** Specimens positive for S100A8, S100A9, and CD15 were counted for each TMA and the percentage of HPV+ and HPV− illustrated. S100A8+S100A9+CD15+ PeCa specimens with positive tumor staging for invasive growth (pT1b-4) **(D)** and metastases (pN1-3) **(E)** depending on their HPV status. As calculated by Fisher's exact test, significant differences are indicated with p ≤ 0.05 (*).

We previously identified a CXCL8-dependent mechanism of neutrophil chemotaxis with HPV^+^CD15^+^ characterizing PeCa with a higher staging regarding invasion and metastasis (16). Here, a remarkable amount of PeCa specimens with infiltrating cells intensively stained for S100A8 and S100A9 was observed (**Figure 2A**). While, in the adjacent normal foreskin, only occasionally S100A8^+^S100A9^+^ immune cells were observed, this dramatically changes in PeCa specimens. Specimens with partially high numbers of intensively stained infiltrating and tumor-intersecting immune cells were identified at the invasion front, at the tumor center, and lymph node metastasis (**Figure 2A**). Reconciling previously published data, a mechanism of elevated expression levels of HPV16 oncoproteins that cause a p63-dependent upregulation of CXCL8, a well-known chemokine for neutrophils, was described (16). This mechanism provided a possible explanation for the infiltration of neutrophils observed in PeCa specimens. For this purpose, the same TMAs as used here were stained for p63 and CD15, with the latter being a marker for neutrophils (16). As described previously (16), we detected a high number of CD15^+^ infiltrating and tumor-intersecting cells in PeCa specimens but not in adjacent normal foreskin (**Figure 2B**) with an overlap of S100A8^+^S100A9^+^ and CD15^+^ immune cells (**Supplementary Figure 3**). Triple-positive (S100A8^+^S100A9^+^CD15^+^) PeCa specimens were with higher-frequency HPV^−^ for the TMA TC and IF, whereas the majority of LM were positive for all four parameters (S100A8^+^S100A9^+^CD15^+^HPV^+^) (**Figure 2C**). S100A8^+^S100A9^+^CD15^+^ PeCa specimens were in tendency rather invasive growing cancers (TMA TC odds ratio = 2.872, TMA IF odds ratio = 3.048) and significantly enriched in the HPV^+^ group of metastasizing PeCa (odds ratio = 8.444, p = 0.0232) (**Figures 2D, E**). Thus, in line with the previous reports (15, 16), our data provide further evidence that patients with PeCa presenting with HPV-related histological subtypes and tumors infiltrated with neutrophils are at a higher risk for the prognosis of more aggressively dedifferentiated growing cancer (**Supplementary Figure 4**) and of lymph node involvement (**Supplementary Figure 5**). Predominantly, three histological subtypes—usual SCC (1), warty-basaloid (11), and basaloid (13)—followed by papillary-basaloid (10) and clear cell carcinoma (14) were characterized by a high amount of S100A8^+^S100A9^+^CD15^+^ cases, particularly if HPV^+^ (**Supplementary Figure 6**), and with lymph node involvement (pN1-3; **Supplementary Figure 5**). Because both S100 subunits mark MDSCs frequently associated with disease progression and treatment failure, these data suggest that the marker profile HPV^+^S100A8^+^S100A9^+^CD15^+^ characterizes patients with PeCa at a higher risk for a worse prognosis.

### Elevated expression CD147 in PeCa is associated with metastasizing cancer

**Figure 3:**
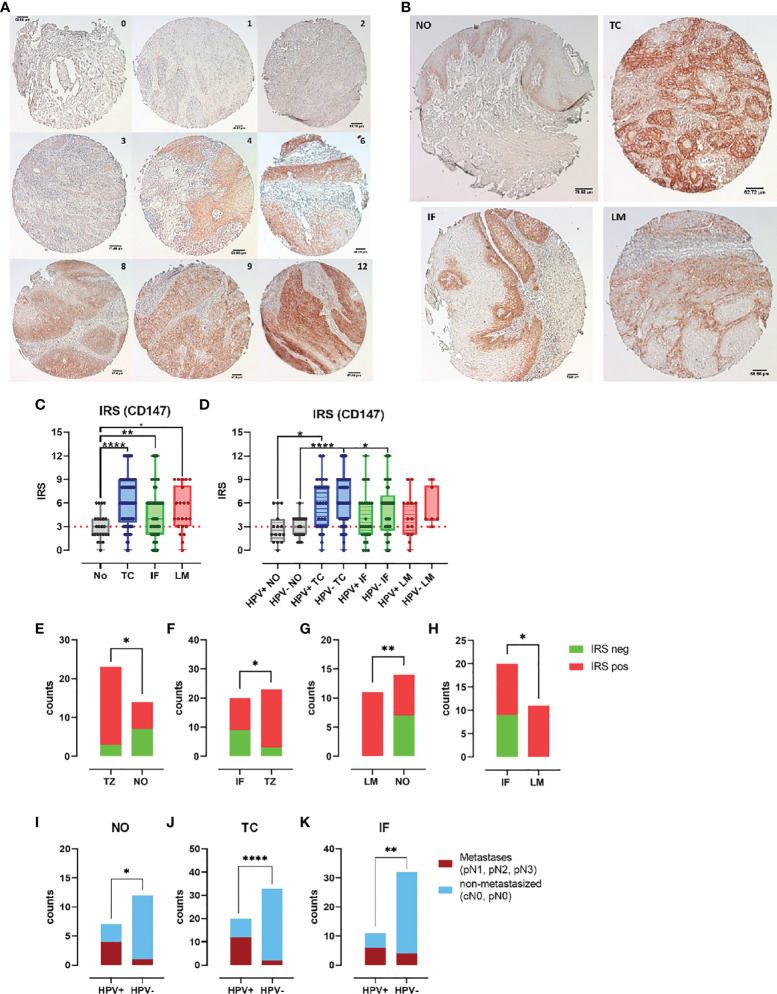
CD147 expression enhanced in PeCa with dissemination potential. **(A)** PeCa specimens were stained using IHC for CD147. According to Remmele and Stegner, IRSs were defined according to staining intensity and the number of stained cells per specimen. **(B)** IHC staining of normal tissue (NO) compared to tumor center (TC), invasion front (IF), and lymph node metastases (LM). **(C)** CD147 IRS for PeCa specimens on the NO, TC, IF, and LM TMA. **(D)** CD147 IRS for HPV^−^ and HPV^+^ PeCa specimens on the NO, TC, IF, and LM TMA. Counts of specimens with positive and negative IRS for CD147 for HPV^+^ specimens comparing the TMA TC vs. NO **(E)**, TC vs. IF **(F)**, LM vs. NO **(G)**, and IF vs. LM **(H)**. Tumor staging of specimens positive for CD147 of HPV^+^ and HPV^−^ PeCa specimens regarding metastasis for the TMA NO **(I)**, TC **(J)**, and IF **(K)**. Significant differences are indicated with p ≤ 0.05 (*), p ≤ 0.001 (**), and p ≤ 0.0001 (****), respectively, as calculated by the Brown-Forsythe and Welch ANOVA test in **(C)** and **(D)**, and the Fisher’s exact test in **(E–K)**.

Because both S100 subunits were notably higher expressed in PeCa specimens, particularly at the tumor center, we questioned if the same might be true for the Calprotectin receptor CD147, especially because the previous data already indicated an increased expression in PeCa (44). Slides of the individual TMA reflecting the tumor center (TMA TC, n = 73), the invasion front (TMA IF, n = 69), and corresponding lymph node metastases (TMA LM, n = 22) and adjacent normal tissue (TMA NO, n = 24) were stained for CD147 expression using IHC, and IRSs were determined (**Figure 3A**). Specimens of the PeCa TMA (TC, IF, and LM) displayed a significantly more intense staining (**Figure 3B**) and a higher scoring than that of the TMA NO (TC vs. NO, p < 0.0001; IF vs. NO, p = 0.0043; LM vs. NO, p = 0.0414) (**Figure 3C**). IRSs of the PeCa specimens of the TMA TC were significantly higher than IRSs of normal tissue in the HPV^+^ and HPV^−^ groups (TC HPV^+^ vs. NO HPV^+^, p = 0.0138; TC HPC^−^ vs. NO HPV^−^, p < 0.0001) and of the TMA IF compared to NO in the HPV^−^ group (p = 0.0122) (**Figure 3D**). CD147^+^ PeCa specimens were significantly enriched in the HPV^+^ group at the TC compared to NO (**Figure 3E**) and IF (**Figure 3F**), as well as for the LM compared to the NO (**Figure 3G**) and IF (**Figure 3H**). All HPV^+^ LMs were positive for CD147 (**Figures 3E, F**). Specimens of the TMA TC, IF, and from the adjacent NO that were positive for CD147^+^ had with a higher-frequency metastases in case of an HPV^+^ than HPV^−^ status (N classification pN1-3) (**Figures 3I–K**). Notably, all HPV^+^ LM specimens were positive for S100A8/A9, CD15, and CD147. These results suggest that CD147^+^ marks HPV^+^ PeCa specimens with a higher risk for lymph node metastases. Finally, our data provide evidence that the Calprotectin-CD147-neutrophil axis plays an important role in disease progression particularly in the event of an HPV^+^ status.

### CD147 expression on cancer cell lines correlates negative with ADCC susceptibility

**Figure 4:**
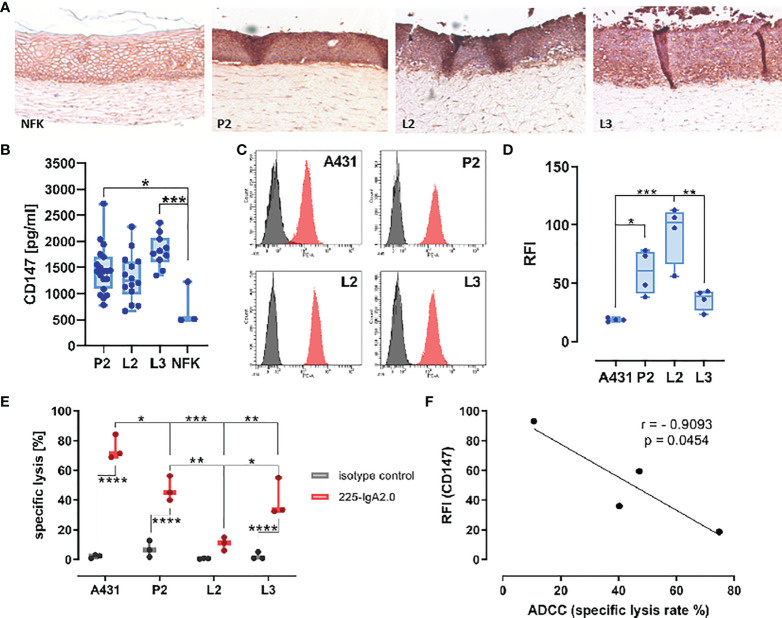
CD147 surface expression correlates negatively with ADCC susceptibility. **(A)** Expression of CD147 was investigated by IHC on FFPE slides of 3D cultures from PeCa cell lines P2, L2, L3, and NFK on a matrix of rat collagen with embedded HFF using mouse anti-human CD147–specific antibody and the Vector goat-anti-rabbit-ImmPRESS DAB Kit. Pictures were recorded with a Leica DMI6000 and ×10 magnification. **(B)** Expression of sCD147 in conditioned media from **(A)** was determined by ELISA. **(C)** Surface expression of mCD147 was investigated by indirect immunofluorescence and flow cytometry using a mouse anti-human CD147–specific antibody and a PE-labeled mIgG-binding protein of the PeCa cell lines P2, L2, L3, and A431. Representative histograms of four independent experiments are shown. **(D)** Relative fluorescence intensity (RFI) of four independent experiments in **(C)** was summarized. **(E)** The capacity of EGFR-directed IgA antibodies (IgA isotype control, 225-IgA2.0, 10 µg/ml) to mediate ADCC of tumor cell lines within 3 h was tested by Calcein release assay using freshly isolated neutrophils (E:T ratio 40:1). A431 served as positive control (30). Calcein-loaded target cells in suspension were simultaneously treated with antibodies and neutrophils for 3 h and fluorescence measured in the supernatant. **(F)** Pearson correlation of mCD147 surface expression on tumor cells in C and specific lysis of the tumor cells in **(D)**. Data are presented as box and whisker plot showing all data points and min and max as “CD147 in [pg/ml]” in **(B)**, as “RFI” in **(D)**, as “specific lysis %” as mean ± SEM in **(E)** of three independent experiment runs in triplicates. Significant differences are indicated by asterisks for p ≤ 0.05 (*), p ≤ 0.01 (**), p ≤ 0.001 (***), and p ≤ 0.0001 (****) as calculated by one-way ANOVA, in **(E)** with two-way ANOVA with Tukey’s multiple comparison test. NFK, normal foreskin keratinocytes; HFF, human foreskin fibroblasts.

Next, we evaluated the expression of CD147 by HPV-positive PeCa cell lines using organotypic 3D raft cultures. These composite of HFF embedded in extracellular matrix with keratinocytes or tumor cells seeded on top at the air-liquid interface that allows them to grow to a multilayered epithelium. This cell culture model creates an environment that mirrors inter-cellular regulatory networks, thus enabling studies of the expression of CD147 in a more physiological context. IHC staining for CD147 on formalin-fixed paraffin-embedded (FFPE) slides of these 3D cultures revealed a more intense staining of HPV-positive PeCa cell lines than that of NFK (**Figure 4A**). Because this approach does not differentiate between the soluble and membranous form of CD147, we tested the conditioned media from these 3D cultures for the release of soluble CD147 (sCD147). We recently identified sCD147 as upregulated in the same HPV-positive PeCa cell lines using a cytokine array and conditioned media from monolayer culture (16). In line with this, media conditioned by 3D cultures of PeCa cell lines contained notable higher amounts of sCD147 than those of NFK (P2 vs. NFK, p = 0.0210; L3 vs. NFK, p = 0.0005; **Figure 4B**). Next, we tested for the expression of surface membranous CD147 (mCD147) expression using indirect immunofluorescence and flow cytometry (**Figures 4C, D**). All three HPV-positive PeCa cell lines expressed higher surface levels of mCD147 than the cell line A431, which was used as HPV-negative control tumor cell line, particularly P2 and L2 (P2 vs. A431, p = 0.0199; L2 vs. A431, p = 0.0002). Notably, the PeCa cell line L2 expressed higher amounts than the cell lines P2 and L3 (L3 vs. L2, p = 0.0019). We described previously that the PeCa cell lines display a different susceptibility to IgA-dependent neutrophil-mediated tumor cell killing (16) and thus questioned whether the amount of mCD147 would be predictive for this. We investigated the capacity of the EGF receptor (EGFR)–directed engineered IgA2 antibody 225-IgA2.0 to engage freshly isolated neutrophils for antibody-dependent cell-mediated cytotoxicity (ADCC) of the PeCa cell lines and A431 cells [**Figure 4E**; re-evaluated from (16)]. The IgA-dependent neutrophil-mediated killing was most effective using the A431 cells, followed by PeCa cell lines P2 (A431 vs. P2, p = 0.0223) and L3 (A431 vs. L3, p = 0.0065) as target cells, whereas the cell line L2 (A431 vs. L2, p = 0.0001) was not lysed. There was a significant difference to the isotype control in case of A431, P2, and L3 (p < 0.0001) but not for L2 (**Figure 4E**). The specific lysis rates for P2 and L3 were significantly higher than for the PeCa cell line L2 (P2 vs. L2, p = 0.0047; L3 vs. L2, p = 0.0156). Thus, mCD147 expression displayed a contrary pattern to the specific lysis rate and correlated negatively with the efficacy of the 225-IgA2.0 to engage neutrophils for tumor cell killing (Pearson, r = −0.9093, p = 0.0454), whereas the soluble variant did not (Pearson, r = −0.6387, p = 0.3613) (**Figure 4F**). Our results suggest that HPV-positive PeCa cell lines expressed higher amounts of CD147 than HPV-negative cells and that CD147 could display a critical factor for neutrophil-centered immunotherapeutic approaches.

## Discussion

PSCC is a malignant disease of the male urogenital tract with rising incidence rates. High age-standardized incidence rates (ASRs) were calculated for countries of South America and Africa, with a Brazilian hot spot in Maranhão with an ASR of, meanwhile, 6.15 per 100,000 (**Supplementary Figure 7A**) (10, 12). With an increasing prevalence of HR-HPV infections at mucosal sites, such as penile, vulva, cervix uteri, anus, and tonsils, the annual incidence rates for HPV-related cancers and, thus, for PeCa will rise. Current data from a countrywide cancer registry underline this hypothesis (**Supplementary Figure 7B**) and show similar trends as data from other anatomical sites (45, 46). An extrapolation of the increment in the ASR until 2030 using a linear regression model suggests that the ASR will increase above 2.0 within the next ten years (**Supplementary Figure 7C**) with a calculated HRPC of 3.2%. Thus, with annually rising case numbers for PeCa, there is a particular need for promising markers to stratify patients according to their risk for invasive and disseminating cancer.

Here, we describe novel and potential markers for the stratification of patients with PeCa regarding their risk for lymph node metastases. The Calprotectin subunits S100A8 and S100A9 as well as the receptor CD147 were elevated expressed in PeCa compared to non-malignant tissue, with a markedly higher expression at the tumor core and lymph node metastases. Overexpression of S100A8/A9 has been reported in precursor lesions at other mucosal anatomical sites susceptible to HPV-driven transformation and may display an early event of the oncoprotein-driven carcinogenesis (20, 23, 47–49). The elevated expression of S100 proteins in cutaneous HPV-related lesions and SCC has been confirmed recently as well (17, 50). Together, there is an emerging evidence that an elevated expression of S100A8 and S100A9 could display a pan-HPV type-specific mechanism during the carcinogenesis at different anatomical sites.

**Figure 5:**
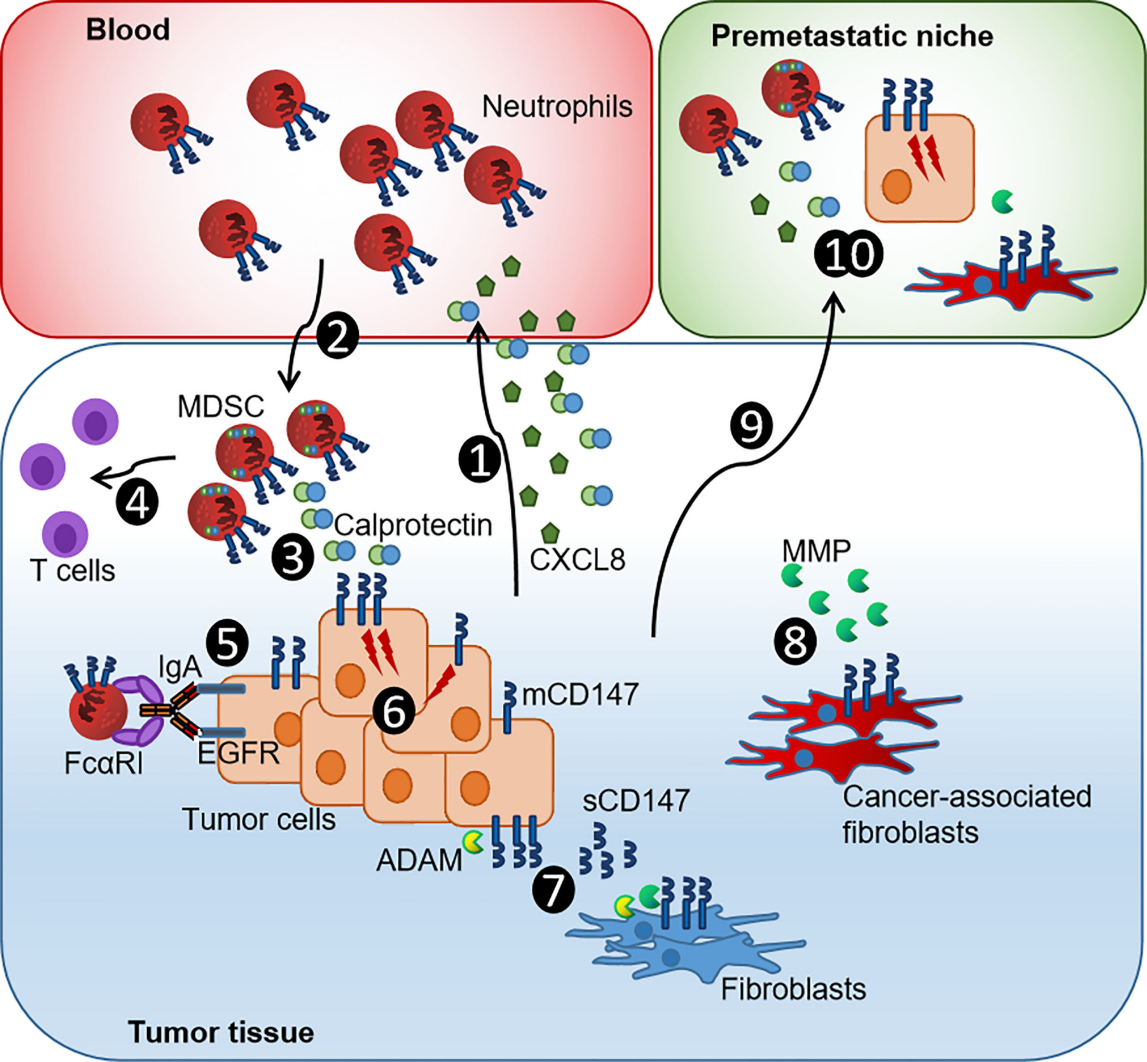
Current model of the CXCL8-Calprotectin-CD147-neutrophil axis in penile tumor progression. (1) PeCa cells release CXCL8 and Calprotectin that promote chemotaxis and infiltration of neutrophils. (2) Infiltrating Calprotectin-positive TME-reprogrammed neutrophil-MDSCs supports tumor growth by releasing growth factors and matrix remodeling enzymes. (3) MDSCs synthesize Calprotectin, supporting an autocrine feedback loop that causes further accumulation of MDSCs. (4) Neutrophil-derived MDSCs have a notable T-cell suppressing activity associated with poor prognosis. (5) The expression of mCD147 impairs ADCC and, thus, the efficacy of immunotherapeutic approaches. (6) The Calprotectin-CD147 axis causes intracellular signaling that enhances the expression of pro-tumorigenic genes and thus promotes proliferation and dissemination of cancer cells. (7) ADAM proteases cleave mCD147 releasing sCD147 that fuels epithelial-to-mesenchymal transition of cancer cells, further boosting invasion and metastasis of cancer cells, and causes a positive feedback loop of sustained CD147-MMP production in fibroblasts. (8) These transform into cancer-associated fibroblasts that, in turn, further promote tumorigenesis by releasing MMP, recruiting further MDSCs and causing an immunosuppressive gene signature in myeloid cells. (9) Tumor-derived factors such as TNF-α, VEGF, and TGF-β induced Calprotectin in distal organs initiating (10) the formation of Calprotectin-CD147-neutrophil MDSC-primed pre-metastatic niches that facilitate the colonization by tumor cells [modified by (53)].

In addition to the expression of S100A8 and S100A9 by the tumor cells themselves, a remarkable influx of S100A8^+^S100A9^+^ immune cells was detected. The TME instructed by cancer and stromal cells, in HPV-negative and HPV-positive cancers, provides soluble factors that recruit myeloid cells including both S100 proteins (1, 2, 16, 45, 51, 52) (**Figure 5**). Once trapped into the TME, myeloid cells can polarize into MDSCs that mediate immunosuppression, support tumor growth, and release angiogenic factors. MDSCs especially at the tumor core and positive for LOX1, a marker for neutrophil-derived MDSCs, have a notable T-cell suppressing activity associated with poor prognosis (41) (**Figure 5**). Neutrophil-derived MDSCs, in turn, release S100A8 and S100A9 to sustain tumor angiogenesis and to create a pre-metastatic niche by facilitating the extravasation and growth of metastasis-initiating cells (1) (**Figure 5**). In conclusion, both S100 proteins drive the carcinogenesis by compromising the myeloid cell compartment with above together with previous data (16), pointing to similar mechanisms during the penile carcinogenesis, particularly during the HPV-driven malignant transformation (**Figure 5**).

However, the role of S100 proteins during HPV-driven carcinogenesis is still not fully understood. There is an increasing evidence for distinct functions of S100A8 and S100A9 if expressed as intracellular and extracellular by tumor or immune cells (1, 2, 20, 53). Intracellular S100A8/A9 controls epithelial differentiation and cleavage of the EGFR (1, 2, 20). Silencing S100A8/A9 in HNSCC cells led to elevated EGFR levels, and a reduced intracellular expression of both S100 proteins was associated with improved survival in HNSCC (20, 23). We observed intracellular expression of S100A8 and S100A9 in HPV-negative and HPV-positive PeCa (**Supplementary Figures 2**, **3**). On the other side, extracellular S100A8/A9 can fuel carcinogenesis by recruiting MDSCs and may play a pivotal role during the amplification of an inflammatory loop in the TME (1, 53) (**Figure 5**). Although these results together with previous data on CXCL8 released by HPV-positive PeCa cell lines point to an increment of neutrophil-MDSC recruitment during (HPV-driven) penile carcinogenesis (**Figure 5**), there might be a distinct role for intracellular expressed S100A8/A9. Respective expression analyses in HPV-positive PeCa cell lines revealed that HPV-positive cell lines were negative for both subunits (**Supplementary Figure 8**). We may hypothesize that our *in vitro* 3D cell culture conditions do not imitate the physiologic conditions completely and that TME-related factors contribute to the *in situ* expression of S100A8/A9 that have not been identified and are not reflected by our culture conditions so far. Several soluble mediators were described to induce S100A8/A9 protein expression including Lipopolysaccharide (LPS), Tumor necrosis factor-alpha (TNF-α), interleukin-1a (IL-1α), interleukin-1b (IL-1β), interleukin-10 (IL-10), interleukin-22 (IL-22), Vascular endothelial growth factor-A (VEGF-A), and Tumor growth factor beta (TGF-β) with LPS indicating a bacterial involvement together with a suggested activation of NFκB and Signal transducer and activator of transcription 3 (STAT3)-based signaling pathways (53). More recent investigations targeting the role of neutrophil subsets during carcinogenesis suggested that distinct subsets, of naturally occurring and TME-induced subsets, could display an increased capacity to support cancer progression and to upregulate S100A8/A9 expression (1, 8). More detailed analyses on the modes of action of S100A8/A9, the putative role of p63 in this context, and how a chronically inflammatory TME supports the penile carcinogenesis may result in the discovery of new therapeutic targets.

The receptor for S100A9 and Calprotectin, CD147, was elevated, as expressed in PeCa specimens. Similar to both S100 subunits, CD147 displayed an elevated expression especially at the tumor core and marked with a higher-frequency disseminating cancer. An elevated expression of CD147 in HPV-related cancers as well as a correlation with Ki-67–positive proliferating cancer cells, T stage, and worse overall survival has been reported previously (31, 32, 44, 54–56) but with limited sample size and high uncertainty in the results for PeCa. CD147 expression is elevated expressed in cells expressing HPV16 oncoproteins, suggesting that viral oncoproteins may act as a direct or indirect inductor of CD147. Elevated expression of CD147 predicts poor prognosis and resistance to radiotherapy and chemo-radiotherapy (31, 32, 54, 55). In a recent large-scale study on multiple cancer types, the expression of CD147 was significantly elevated expressed in 24 of the 31 cancer types, related to immune infiltration, poor outcome regarding overall and disease-free survival, as well as predictive for (immuno-) therapeutic response (56). The study confirmed the elevated expression of CD147 in PeCa using multiplex immunofluorescence staining on TMA. Moreover, the authors identified a high level of CD147^+^ M2 macrophages in cervical cancer, another highly HPV-related entity, suggesting that monocyte- and neutrophil-derived MDSCs can express CD147. Although the infiltration of MDSCs expressing S100A8 and S100A9 has been closely related to tumor stage, lymph node metastases, and poor prognosis (57), there is a rising evidence that CD147^+^ myeloid cells are key players in HPV-driven cancers. However, the underlined technique did not differentiate between the membranous and soluble version of CD147. Shedding of CD147 from the surface is mediated by MMP and ADAM proteases with the soluble version associated with tumor growth, metastasis, and chemoresistance (58–61) (**Figure 5**). Although our results, so far, do not support a critical role of sCD147 for the susceptibility of cancer cells to neutrophil-mediated ADCC, the soluble isoform converts quiescent fibroblasts into cancer-associated fibroblasts and promotes the epithelial-to-mesenchymal transition of cancer cells, further boosting invasion and metastasis of cancer cells (30) (**Figure 5**). Evaluating the detailed molecular mechanisms of the CD147-fueled and HPV-driven tumorigenesis by future studies deciphering the individual contribution of membranous and soluble CD147 will provide enormous benefits in developing new treatments for HPV-associated entities, such as PeCa.

Faced with a continuous majorly HPV-related increment in incidence rates, markers for improved patient stratification are highly needed. Parameters describing the dissemination potential of the primary tumor may help to identify patients at a higher risk for more aggressive cancers and lymph node metastasis. Here, we described novel markers—S100A8, S100A9, and CD147—which in combination with our recent data on CD15 (16), point to a prominent role of the Calprotectin-CD147-neutrophil axis in the progression of penile cancer that may have additional implications for immunotherapeutic approaches, requiring neutrophils for their efficacy. Respectively, therapeutic strategies may improve the outcome of cancer treatments by interfering with these carcinogenesis accelerating regulatory cascades.

## Data availability statement

The raw data supporting the conclusions of this article will be made available by the authors, without undue reservation.

## Ethics statement

The studies involving human participants were reviewed and approved by Ärztekammer des Saarlandes, Saarbrücken, Germany. The patients/participants provided their written informed consent to participate in this study.

## Author contributions

TM, AZ, MH, IB, FB, and SL: resources, data curation, formal analyses, investigation, visualization, methodology, and software; TM and SL: conceptualization; TM, AZ, KJ, and SL: validation and supervision; OK, VM, PL, AP, AH, C-IG, HL, HW, CN, HK, and SS: resources and data curation; TM, IB, MH, CN, HK, KJ, and SL: interpretation and writing—review editing; SL: funding acquisition, writing—original draft, and project administration. All authors contributed to the article and approved the submitted version.

## Funding

This work was supported by the German Research Foundation (DFG, Lo 1853/1-2) and intramural funding (HOMFOR, start-up promotion, Young Investigator Grant).

## Acknowledgments

We gratefully acknowledge the excellent technical assistance from Katrin Thieser and Barbara Best from the Institute of Virology and Alexander Vogt from the Department of Urology at the Saarland University Medical Center. We are very thankful for the help with the microscope (Imager M2, Zeiss) by Prof. Dr. Gabriela Krasteva-Christ (Institute of Anatomy and Cell Biology, Saarland University, Homburg, Germany).

## Conflict of interest

The authors declare that the research was conducted in the absence of any commercial or financial relationships that could be construed as a potential conflict of interest.

The reviewer LM declared a shared affiliation, with no collaboration, with some of the authors AH and C-IG to the handling editor at the time of review.

## Publisher’s note

All claims expressed in this article are solely those of the authors and do not necessarily represent those of their affiliated organizations, or those of the publisher, the editors and the reviewers. Any product that may be evaluated in this article, or claim that may be made by its manufacturer, is not guaranteed or endorsed by the publisher.
