# Supplementary material for: The prominent role of the S100A8/S100A9-CD147 axis in the progression of penile cancer
Source: Front Oncol. 2022 Oct 11;12:891511. doi: 10.3389/fonc.2022.891511 (PMC9592847; doi:10.3389/fonc.2022.891511)

**Supplementary Material:**

**Supplementary Table 1: Patient and disease characteristics:** Total number, age distribution and tumor staging (TNM classification of the 8^th^ edition) of specimens tested by TMA staining.


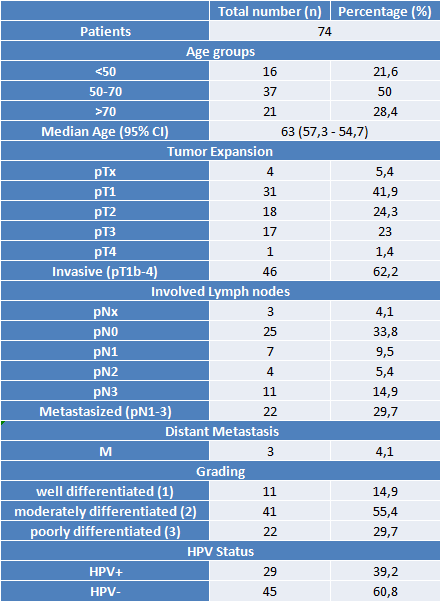


**Supplementary Figure 1: Staining patterns for S100A8 in PeCa specimens:** IHC for S100A8 of non-malignant tissue (A), HPV-positive (B) and -negative (C) PeCa specimens. Representative images are displayed.


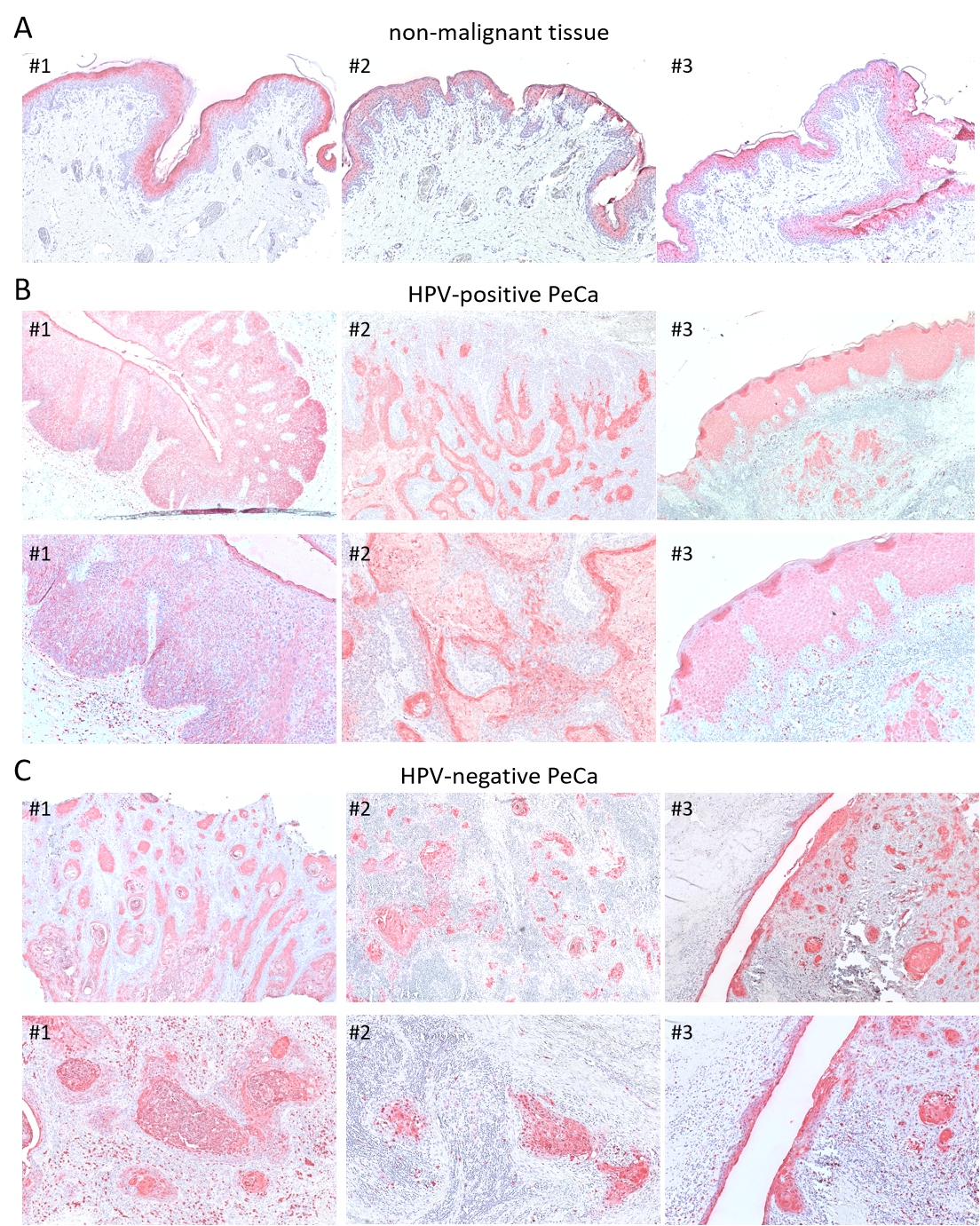


**Supplementary Figure 2: Staining patterns for S100A9 in PeCa specimens:** IHC for S100A9 of non-malignant tissue (A), HPV-positive (B) and -negative (C) PeCa specimens. Representative images are displayed.


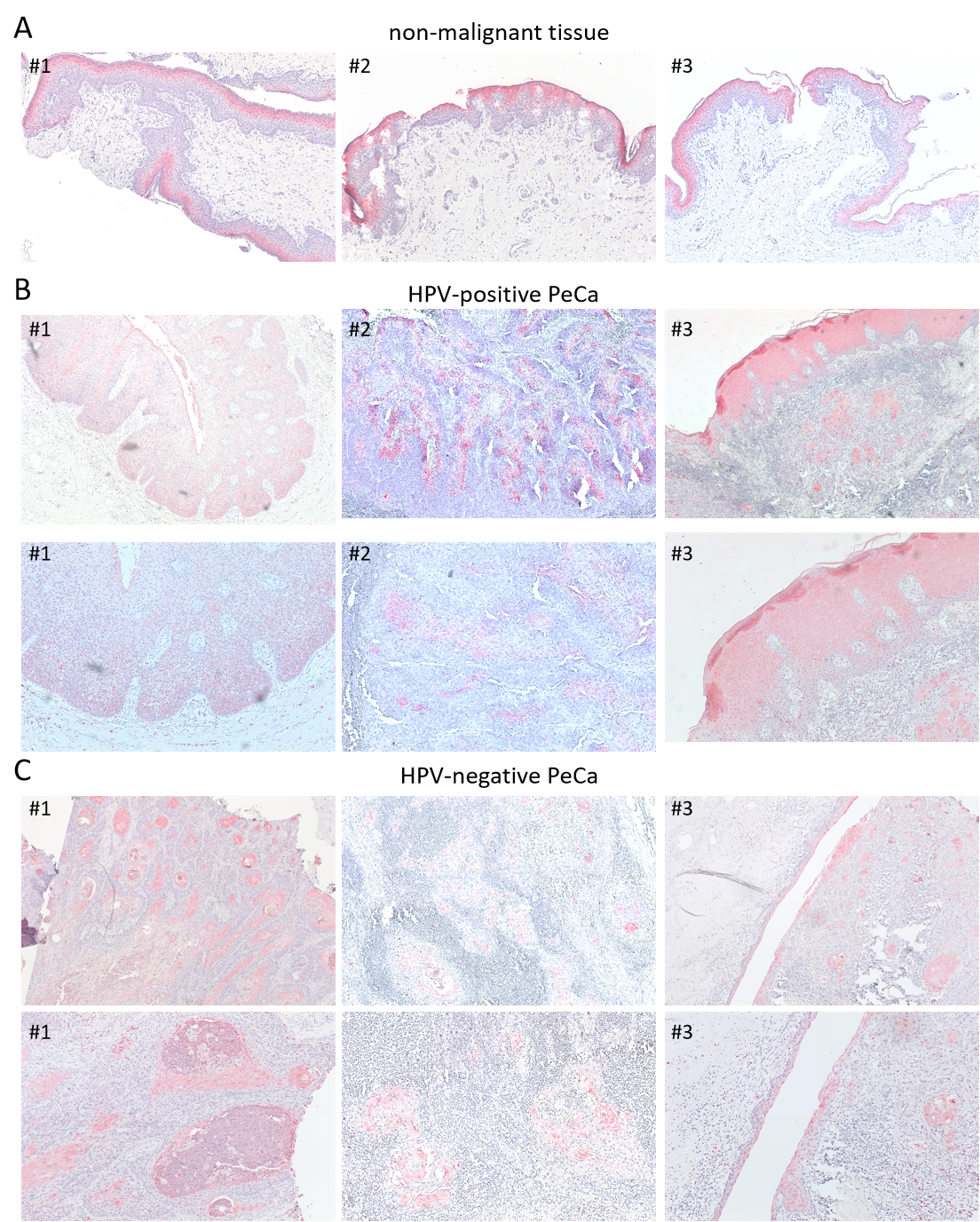


**Supplementary Figure 3: Infiltrating S100A8^+^S100A9^+^ immune cells in PeCa specimens:** TMA containing PeCa specimens were stained for S100A8 and S100A9 and previously for CD15 (16) by IHC. Images display representative pictures of the three TMAs reflecting invasion front (IF), tumor center (TC) and lymph node metastasis (LM) with partial overlap of CD15 with S100A8 and S100A9 staining.


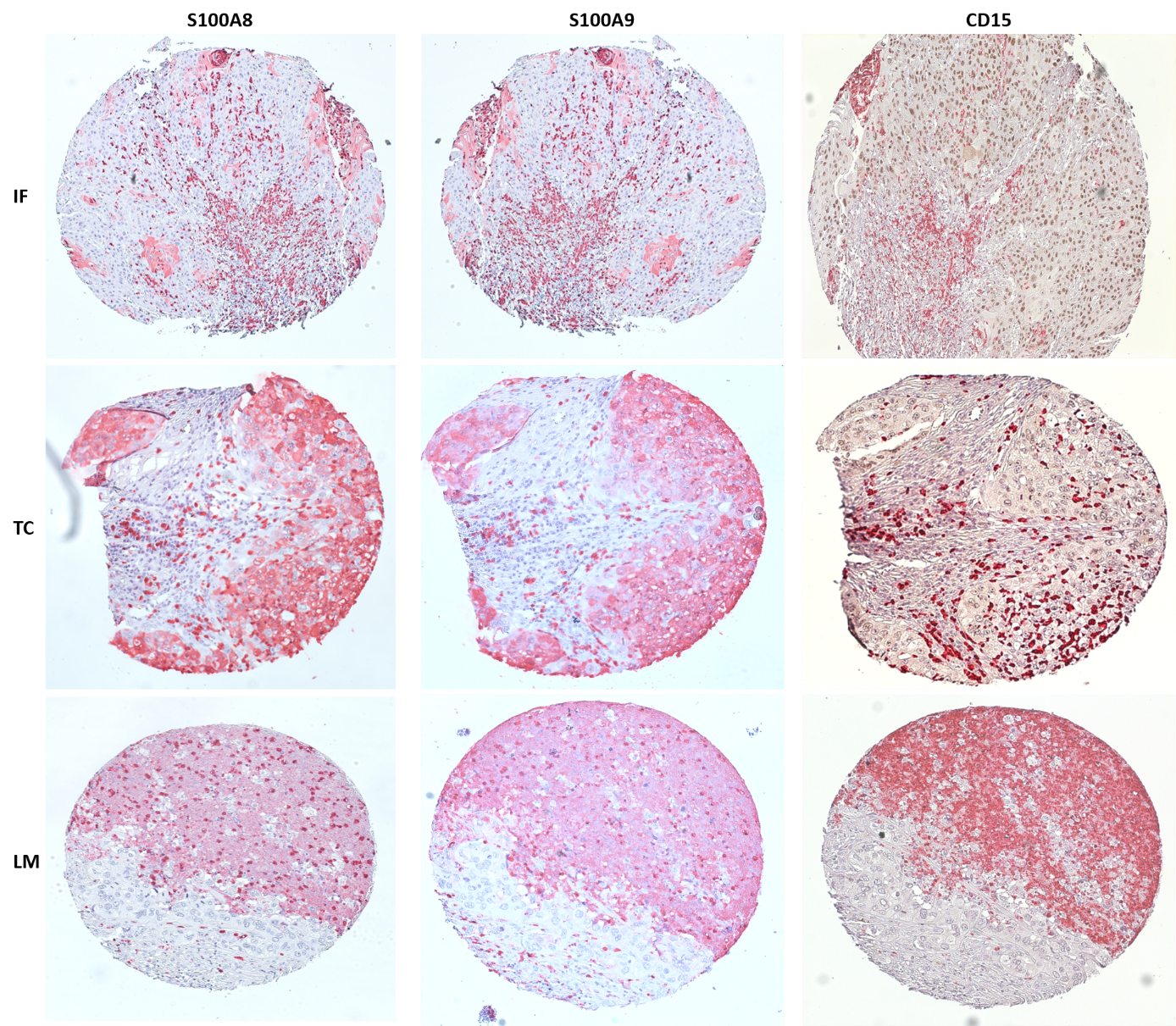


**Suppl. Fig. 4: Staging regarding differentiation of PeCa specimens with positive IRS for S100A8, S100A9, CD15 and positive HPV status with histological subtype:** PeCa specimens of each TMA with positive IRS for S100A8 (A), S100A9 (B), HPV (C), and combined CD15 (D) were stratified according to the histological subtype and grading regarding differentiation.


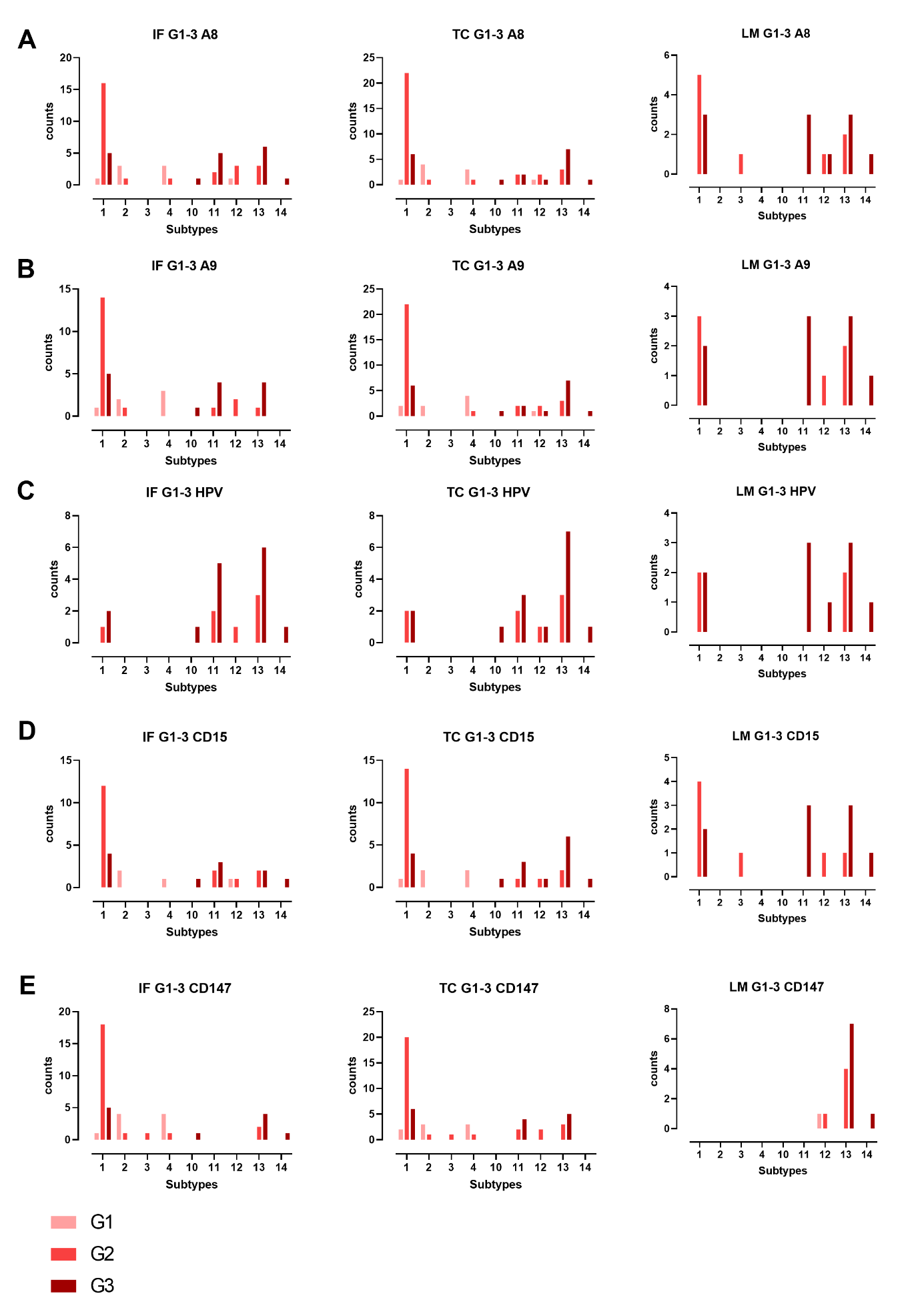

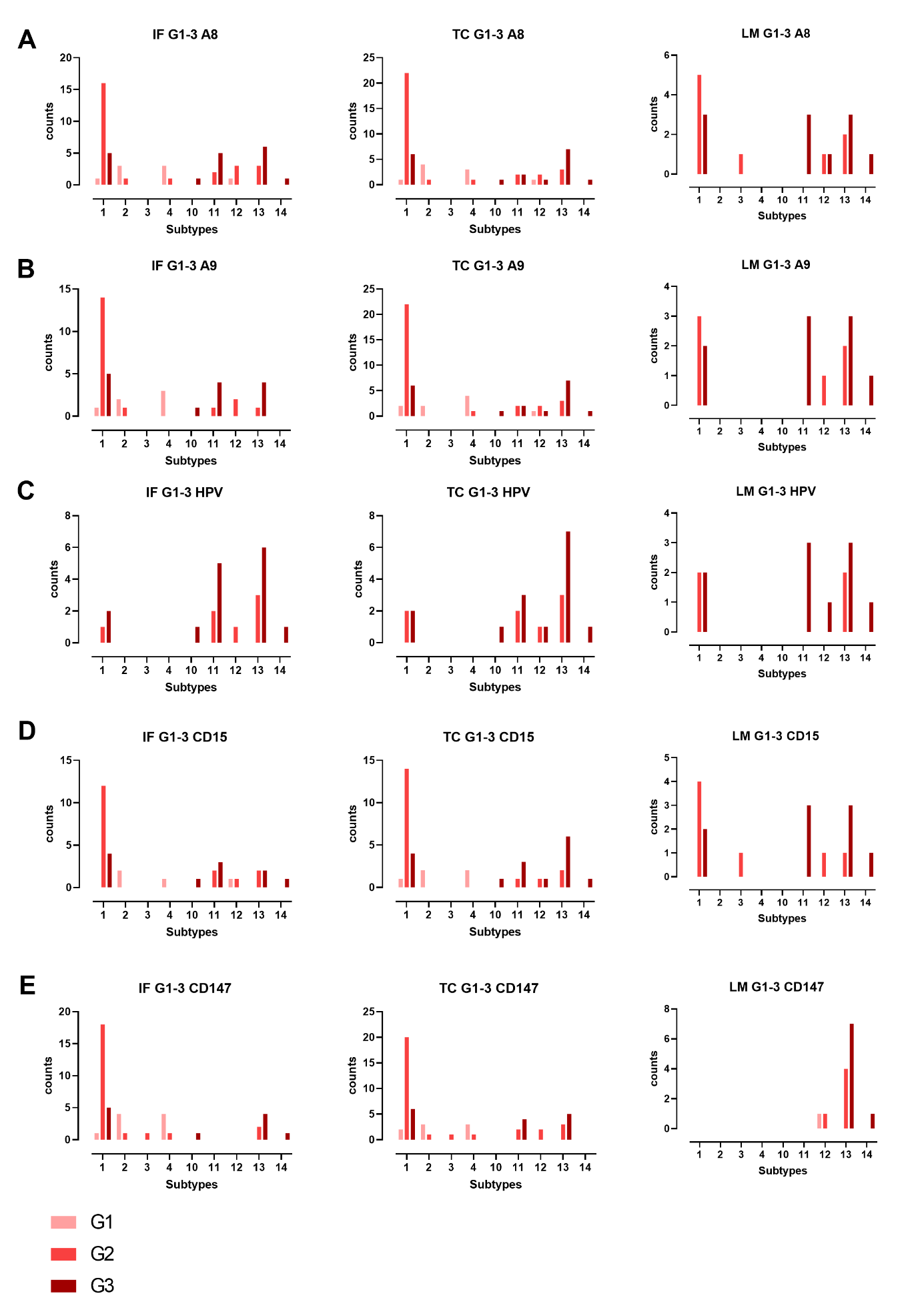


**Suppl. Fig.5: TNM classification of PeCa specimens with positive IRS for S100A8, S100A9, CD15 and positive HPV status depending on histological subtype:** PeCa specimens of each TMA with positive IRS for S100A8, S100A9 or both, positive for CD15, HPV or both were stratified according to histological subtype and staging for invasive growth and metastases.


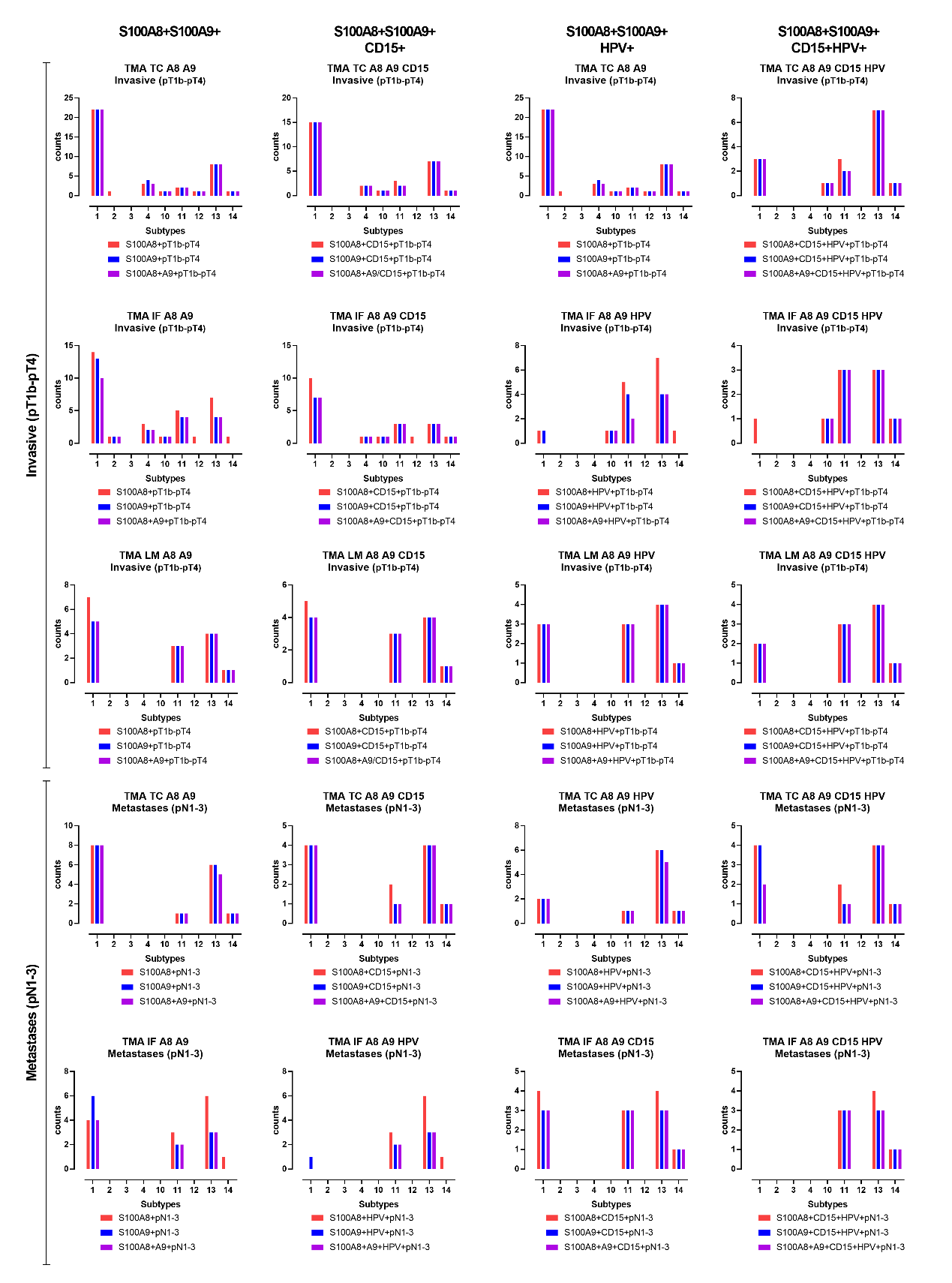


**Supplementary Figure 6: PeCa specimens with positive IRS for S100A8, S100A9, CD15 and positive HPV status depending on histological subtype:** PeCa specimens of each TMA with positive IRS for S100A8, S100A9 or both (A-D), positive for CD15 (C, D), and HPV (B, D) were stratified according to histological subtype.

**
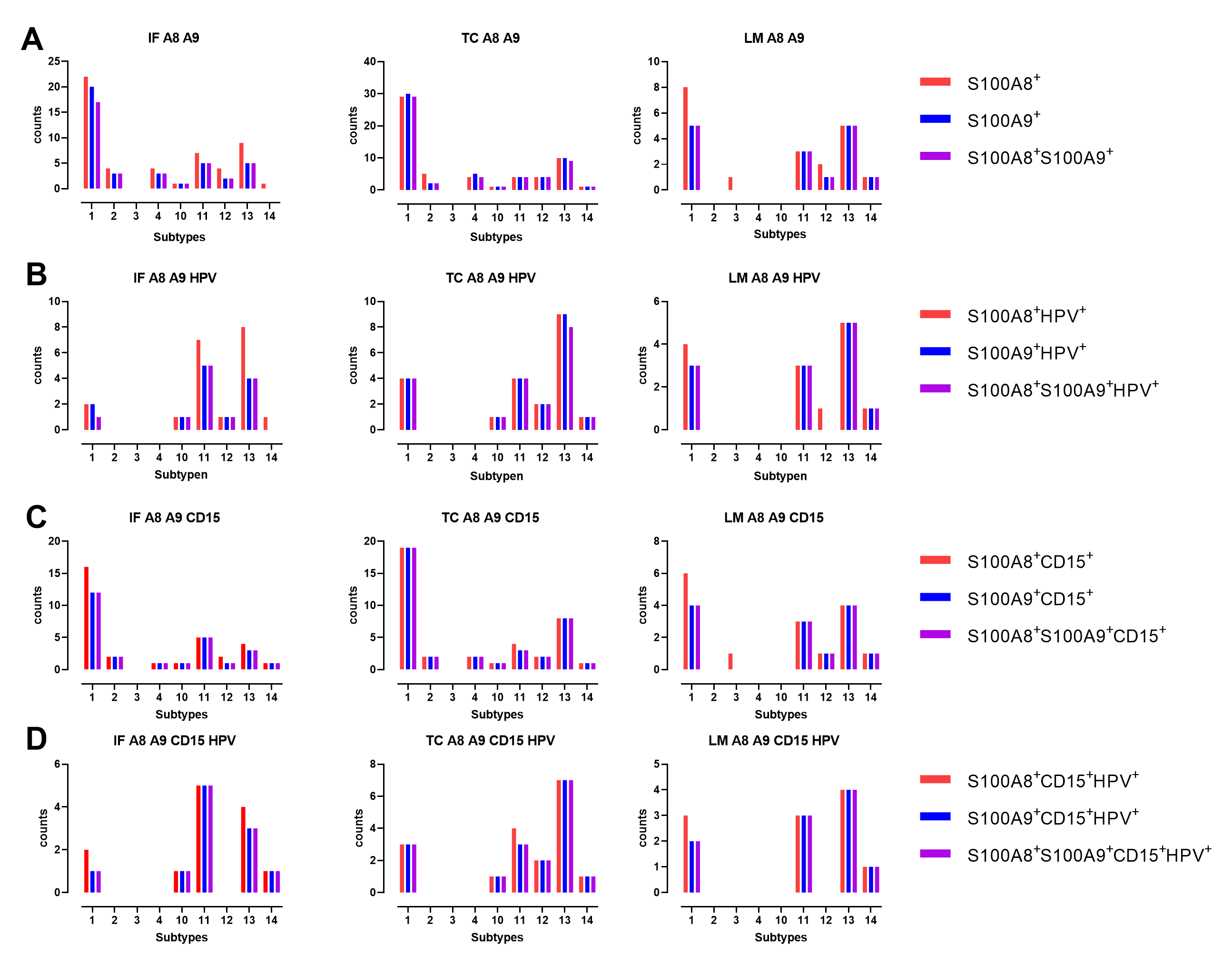
**

**Supplementary Figure 7: Epidemiologic data on PeCa:** A) Age-standardized incidence rate (ASR) of PeCa/100.000 residents worldwide, adapted from (34). B) ASR of PeCa/100.000 residents in Germany from 1999-2016 based on cancer registry data of the RKI (35). C) Linear regression extrapolation model to illustrate the increase of the ASR in Germany until 2030.


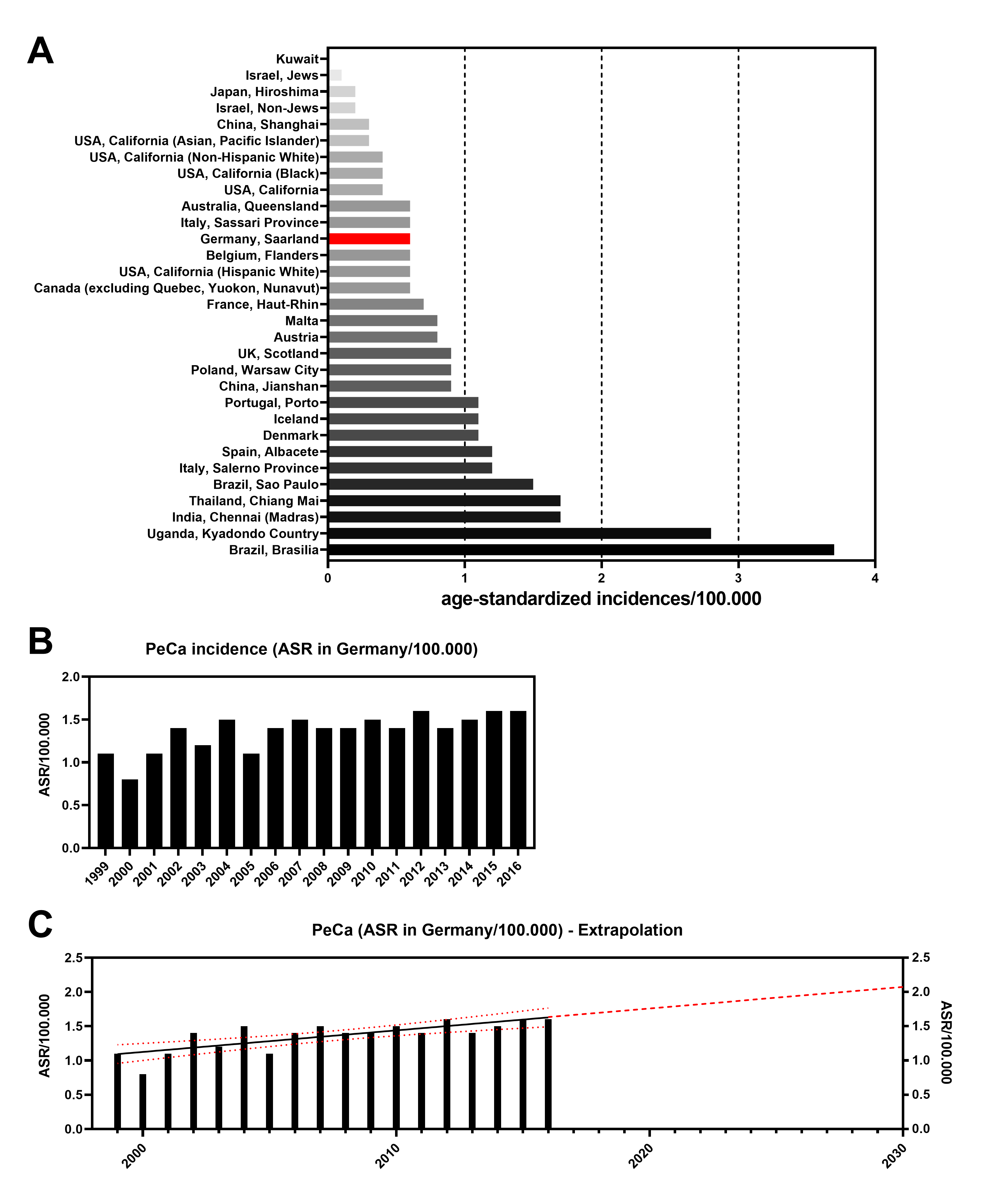


**Supplementary Figure 8: Calprotectin expression in HPV-positive PeCa cells:** Expression of S100A8 and S100A9 was analyzed using IHC and FFPE of organotypic 3D cultures of NFK and PeCa cell lines with the S100A8 (#NBP1-42076, rabbit anti-human S100A8, RRID:AB_2184111, Novus Biologicals, Cambridge, UK) or S100A9 (#sc-20173, rabbit-anti-human S100A9, RRID:AB_2184420, SantaCruz, Heidelberg, Germany) -specific antibodies followed by AP-conjugated anti-rabbit antibody incubation and developed with DAB or AP-substrates (ImmPRESS AP Reagent Kit Alkaline Phosphatase Anti-Rabbit IgG, VECTOR Red Alkaline Phosphatase Substrate Kit, both Vector, Burlingame, CA). Pictures were recorded with 20x magnification, 0.5 cm = 100 µM. Images are representative pictures of five independent experiments of three independent cultures run in duplicates. NFK = normal foreskin keratinocytes. HPV-positive PeCa cells were cultured in organotypic 3D cultures as previously described (16).


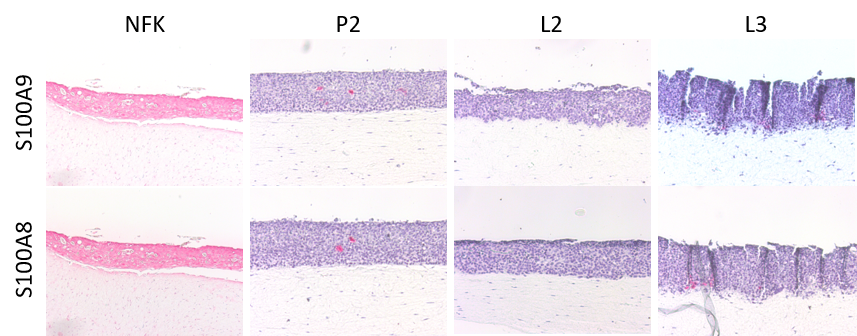

Supplement: Supplementary file 1 [file DataSheet_1.docx]
